# Biomarker Alteration after Neoadjuvant Endocrine Therapy or Chemotherapy in Estrogen Receptor-Positive Breast Cancer

**DOI:** 10.3390/life13010074

**Published:** 2022-12-27

**Authors:** Mengping Long, Chong You, Qianqian Song, Lina X. J. Hu, Zhaorong Guo, Qian Yao, Wei Hou, Wei Sun, Baosheng Liang, Xiao-Hua Zhou, Yiqiang Liu, Taobo Hu

**Affiliations:** 1 Department of Pathology, Peking University Cancer Hospital, Beijing 100142, China; 2 Chongqing Research Institute of Big Data, Peking University, Chongqing 401121, China; 3 Beijing International Center for Mathematical Research, Peking University, Beijing 100871, China; 4 Department of Biostatistics, School of Public Health, Peking University, Beijing 100191, China; 5 Department of Pathology, Alaska Native Medical Center, Anchorage, AK 99501, USA; 6 Department of Breast Center, Peking University Cancer Hospital, Beijing 100142, China; 7 Department of Breast Surgery, Peking University People’s Hospital, Beijing 100044, China

**Keywords:** estrogen receptor, progesterone receptor, Ki-67, neoadjuvant endocrine therapy, neoadjuvant chemotherapy, biomarker

## Abstract

In estrogen receptor (ER)-positive breast cancer, changes in biomarker expression after neoadjuvant therapy indicate the therapeutic response and are prognostic. However, there is limited information about the biomarker alteration caused by neoadjuvant endocrine therapy in ER-positive and human epidermal growth factor receptor 2 (HER2)-negative breast cancer. We recruited ER-positive/HER2-negative breast cancer patients who received neoadjuvant chemotherapy (NCT), neoadjuvant endocrine therapy (NET), or sequential neoadjuvant endocrine-chemotherapy (NECT) at Peking University Cancer Hospital from 2015 to 2021. A total of 579 patients had paired immunohistochemistry information in both diagnostic biopsy samples and post-neoadjuvant therapy surgical samples. Through a paired comparison of the immunohistochemical information in pre-treatment and post-treatment samples, we found that progesterone receptor (PR) expression reductions were more frequent than ER expression reductions (70.8% vs. 35.2%) after neoadjuvant therapy. The percentage of patients who had a decreased Ki-67 index in the post-operative samples was similar in the three groups (79.8% vs. 79.7% vs. 78.4%). Moreover, PR losses caused by NET were related to low baseline PR expression (*p* = 0.001), while we did not find a significant association between PR losses and Ki-67 reductions (*p* = 0.428) or ER losses (*p* = 0.274). All three types of neoadjuvant therapies caused a reduction in ER, PR, and Ki-67 expression. In conclusion, we found that PR loss after NET was only significantly related to low baseline PR expression, and there is no significant difference in the extent of prognostic factor change including Ki-67 and ER between the PR loss and non-loss groups.

## 1. Introduction

Estrogen receptor (ER)-positive breast cancer accounts for about 70% of all breast cancer in which the ER pathway is the driving transcription factor promoting cell proliferation [1]. The systematic therapy for ER-positive breast cancer includes both chemotherapy and endocrine therapy, which can be given in adjuvant and neoadjuvant settings. Neoadjuvant chemotherapy (NCT), neoadjuvant endocrine therapy (NET), and neoadjuvant sequential endocrine-chemotherapy (NECT) have been used in ER-positive breast cancer for almost two decades mostly to downstage tumors before breast-conserving surgery (BCS) [2]. Compared with NCT, NET has less cytotoxicity and is currently applied in older patients and patients with ER-rich tumors which are defined with an “Allred score” greater than 5 [3,4]. There were reports that NECT did not improve the pathologically complete response (pCR) compared with NCT alone in ER-positive and HER2-negative breast cancer [5,6].

The usage of neoadjuvant therapy provides us an opportunity to investigate the biological process caused by the treatment which is mostly characterized by the alteration in the expression of biomarkers, including ERs, progesterone receptors (PRs), and Ki-67. Both ERs and PRs are hormonal receptors which are routinely stained through immunohistochemistry (IHC) according to the ASCO/CAP guidelines [7]. The status of ERs and PRs are not only an indicator for the subtyping of breast cancer but can also work as a therapeutic target of endocrine therapy. Ki-67 is a biomarker for cell proliferation and is also a prognostic marker in breast cancer. Previous studies showed that all three biomarkers would experience a trend of decreased expression in the post-treatment sample for both NET and NCT [8,9,10,11]. Niikura et al. reported that the proportion of patients in which the immunohistochemistry (IHC) status of ERs or PRs turned from positive to negative after NCT were 4.6% and 14% [12]. However, data is currently limited for the NET and NECT groups. The reduction in post-treatment Ki-67 expression was shown to be increased with longer use of endocrine therapy [10]. Moreover, it has been shown that for NET, the post-treatment expression of biomarkers, including ERs and Ki-67, combined with clinical information can be integrated as a scoring system, named preoperative endocrine prognostic index (PEPI), and, hence, could serve for outcome predicting [13,14].

Thus, the change in biomarker expression after neoadjuvant therapy in ER-positive breast cancer can both indicate the biological response and serve as a potential prognostic marker. However, studies comparing the biomarker change in NET, NCT, and NECT were limited. In this study, we performed a comparison of the changes in biomarker expression, including ERs, PRs, and Ki-67, after NET, NCT, and NECT treatment as a retrospective study in an attempt to elucidate the underlying biological mechanisms.

## 2. Materials and Methods

### 2.1. Study Population

**Figure 1:**
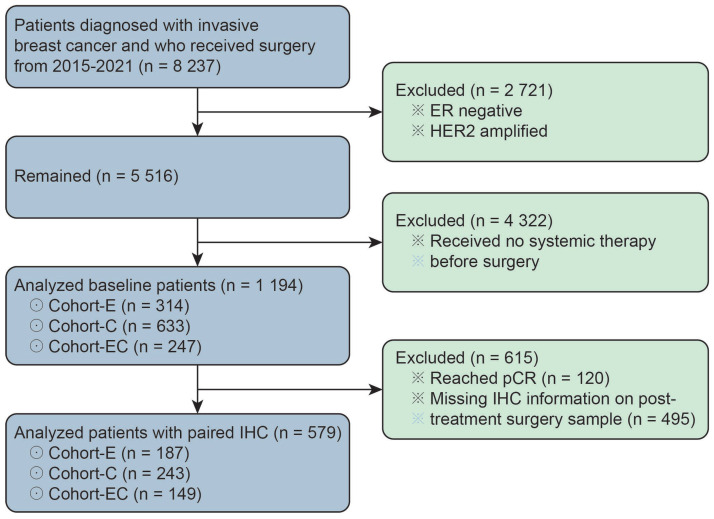
The patient population for baseline information analysis and paired IHC information analysis. ER represents estrogen receptor; pCR represents pathological complete response; IHC represents immunohistochemistry.

This study was approved by the Peking University Cancer Hospital’s Ethics Committee. The pathology database in Peking University Cancer Hospital was queried for breast apocrine carcinomas diagnosed between 2015 and 2021. A total of 1194 luminal breast cancer patients who received neoadjuvant therapy were recruited for our study. All of the included patients were ER-positive and HER2-negative as diagnosed through CNB biopsy. The ER positivity was defined by ≥1% of tumor cells demonstrating positive nuclear staining through IHC. HER2 negativity was defined by IHC score of 0/1 + or IHC score of 2 + with negative amplification through fluorescence in situ hybridization. Among them, 579 patients (48.5%, 579/1194) had paired IHC information on ERs, PRs, Ki-67, and EGFRs in both core needle biopsy samples before neoadjuvant therapy and surgical samples after neoadjuvant therapy. The enrolled 1194 patients were classified into three groups, namely Cohort-C (*n* = 633), Cohort-E (*n* = 314), and Cohort-EC (*n* = 247), respectively, according to the type of neoadjuvant therapy they received (Figure 1). By definition, patients in Cohort-C and Cohort-E received only chemotherapy or endocrine therapy before surgery, while patients in Cohort-EC received chemotherapy in combination with endocrine therapy in the neoadjuvant setting.

### 2.2. Pathological Evaluation

The IHC staining procedure was conducted as described in our previous study [15]. Antibody information was as following: ER (SP1, Roche, 1 µg/mL), PR (1E2, Roche, 1 µg/mL), HER2 (4B5, Ventana, 6 µg/mL), Ki-67 (M1B1, Zhongshan Jinqiao, working concentration), and EGFR (EP22, Jinbiaoyatu, working concentration). The number of patients with different expression levels of ER, PR, EGFR, and HER2 were listed in Table 1 and Table 2. The Allred score of ERs and PRs was evaluated as in the previous studies [14,16,17]. An Allred score of 0 to 2 was considered negative, and scores ranging from 3 to 8 were positive. Moreover, An Allred score of 6–8 was defined as ER/PR-rich, while a score of 3–5 was defined as ER/PR-moderate. The Ki-67 percentage score was defined as the percentage of positively nuclear-stained cells divided by the total number of malignant cells scored. When the staining was homogeneous across the sample, a global Ki-67 was used, and, for heterogeneous staining, Ki-67 was counted in the hotspot regions.

### 2.3. Statistical Analysis

All the statistical analyses were performed using R statistical software (version 4.1.2). For analyses in Table 1 and Table 3, the *“finalfit”* package was used. For matched-pair analyses in Table 2, paired t-test was used for numerical variables including Ki-67, and McNemar’s chi-squared test was used for categorical variables, including ER Allred scores, PR Allred scores, and HER2 values. *p*-values were calculated as two-sided, with statistical significance being declared for *p*-values less than 0.05.

## 3. Results

### 3.1. Patient Selection and Baseline Clinicopathological Information

The enrolled 1194 luminal breast cancer patients who received neoadjuvant therapy belonged to the three groups named Cohort-C, Cohort-E, and Cohort-EC, respectively. The patients in the Cohort-E group were relatively older and had a less advanced histological grade and lower Ki-67 than the other two groups (Table 1). Compared to the patients in the Cohort-C group, the patients who received endocrine therapy in the Cohort-E and Cohort-EC groups were more likely to be ER- or PR-rich, which was defined by an Allred score greater than 5. Patients in Cohort-E tended to have a longer endocrine therapy use duration (9.9 months vs. 8.5 months) and more frequent aromatase inhibitor (AI) usage (90.8% vs. 83.8%) compared with patients in Cohort-EC. The mean length of endocrine therapy in our study was longer than the current conventional NET length, ranging from 3 to 6 months [18,19].

### 3.2. Alteration of ER, PR, and Ki-67 Expression after Neoadjuvant Therapy

**Figure 2:**
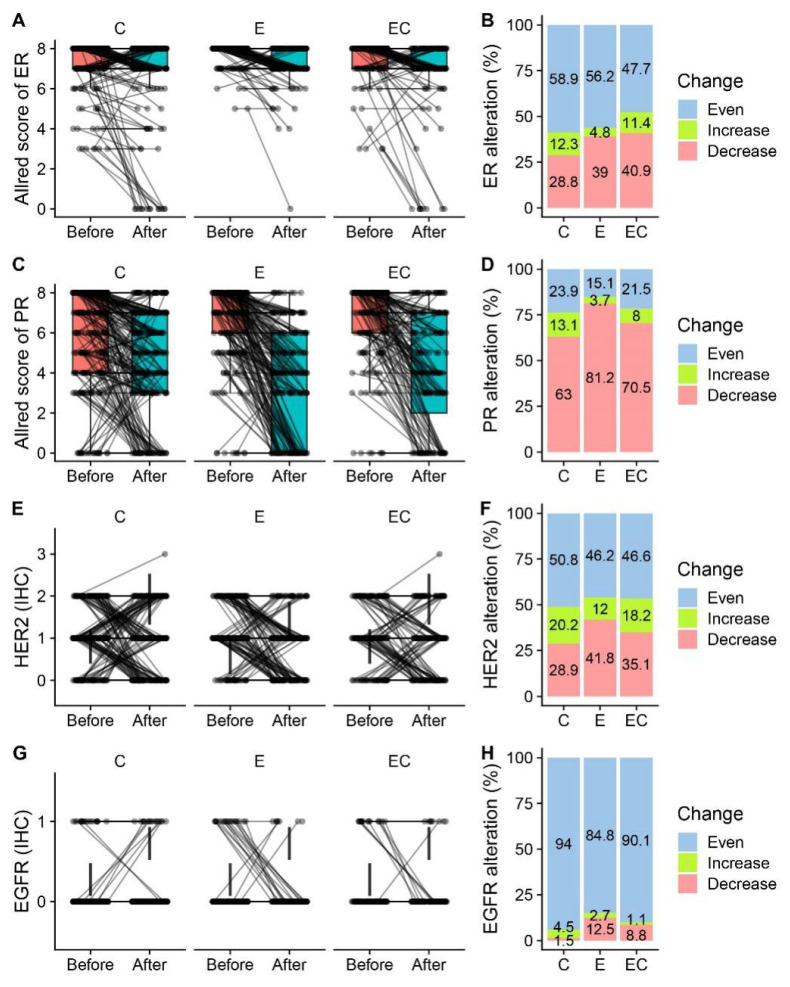
Alteration of ER and PR expression in before- and after-therapy samples by treatment group. (**A**,**C**,**E**,**G**) Paired box plot presenting paired ER Allred scores, PR Allred scores, and HER2 and EGFR values in before-therapy and after-therapy samples in neoadjuvant chemotherapy Cohort-C, neoadjuvant endocrine therapy Cohort-E, and neoadjuvant endocrine therapy combined with chemotherapy Cohort-EC. (**B**,**D**,**F**,**H**) Percentage of patients who had increased, decreased, and unchanged ER Allred scores, PR Allred scores, and HER2 and EGFR values in the three treatment groups.

**Figure 3:**
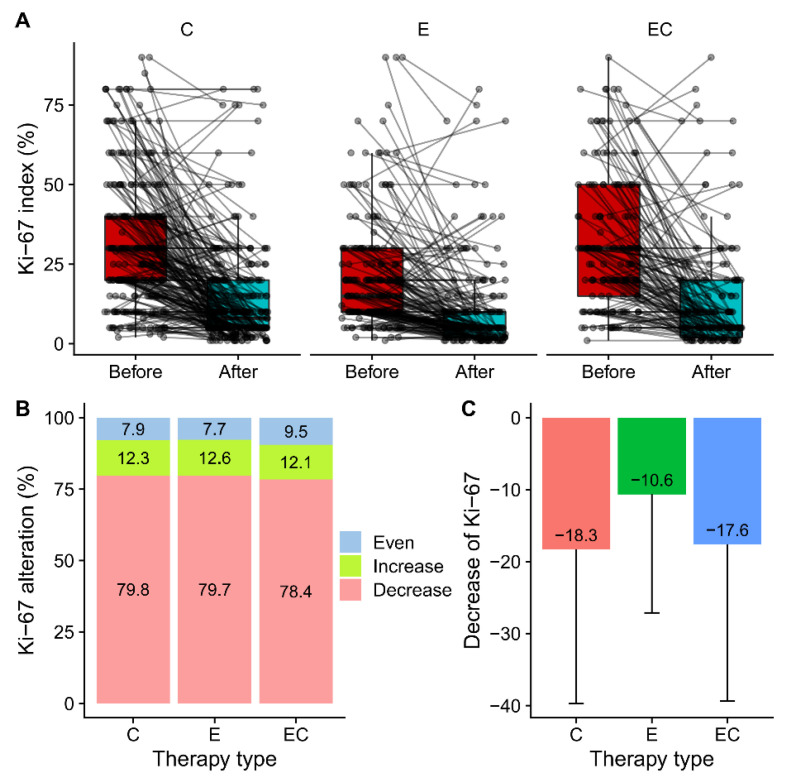
Alteration of Ki-67 in before and after therapy samples by treatment groups. (**A**) Paired box plot presenting paired Ki-67 in before therapy and after therapy samples in Cohort-C, Cohort-E and Cohort-EC. (**B**) Percentage of patients who had increased, decreased and unchanged Ki-67 in three treatment groups. (**C**) Suppression of Ki-67 by treatment in three groups calculated by subtracting post-therapy value with pre-therapy value.

Among all the patients who received neoadjuvant therapy, 48.5% (579/1 194) of them had paired IHC information in both the pre-treatment samples and post-treatment samples. Alterations in ERs, PRs, Ki-67, EGFRs, and HER2 were presented and compared (Figure 2 and Table 2). Among the five biomarkers, ERs, PRs, and Ki-67 all had significant suppression in all three groups, while HER2 had no alteration in any group. The reduction in PR expression after therapy was more frequent than that in ER expression in all the patients (70.8% vs. 35.2%). For ERs, more than half of the patients had unaltered Allred scores in the Cohort-E and Cohort-C groups. The percentage of patients with decreased ER scores in the C, E, and ET groups was 28.8%, 39%, and 40.9%, respectively, suggesting a potential combined effect of chemotherapy and endocrine therapy on the suppression of ER. Twenty-two patients (22/579, 3.8%) became ER-negative in the post-operative samples. However, the decrease of PRs was most significant in the Cohort-E group, suggesting a dominant role of endocrine therapy in the suppression of PRs. The alteration of the HER2 status was not uncommon with about half of the samples showing an altered status among the range of 0, 1+, and 2+ (Figure 2E,F). Interestingly, two patients (2/579, 0.3%) tested HER2 positive with an IHC score of 3 + in the post-neoadjuvant-therapy samples. The alteration of the HER2 status from negative to positive after neoadjuvant therapy was also observed at a higher frequency in a previous study [12]. As for the alteration of EGFRs, there was only a limited number of patients who had paired IHC information. The majority of them showed an unchanged EGFR status in the post-neoadjuvant-therapy samples (Figure 2G,H). The percentage of patients who had decreased Ki-67 in the post-operative samples was similar in the three groups (79.8% vs. 79.7% vs. 78.4%) (Figure 3A,B). Meanwhile, there were about 12% of patients that had increases in Ki-67 in the three groups, which was inconsistent with the previous studies [3]. The decrease in the mean amount of Ki-67 was smaller in the Cohort-E group than that in the Cohort-C and Cohort-EC groups (Figure 3C). However, since the baseline Ki-67 in the Cohort-E group was the lowest, the post-operative Ki-67 in the Cohort-E group was still the lowest among the three groups (Table 2). To look at the biomarker alterations in the different subgroups, we divided the patients in the NET Cohort according to age (≤60 years and >60 years) and therapy length (≤6 months and >6 months). The percentage of patients with ER, PR, or Ki67 alterations was listed in Table 3. No significant difference was seen between the two age groups or between the two therapy-length groups.

### 3.3. PR Loss Was Associated with Baseline PR Score but Not with Ki-67 Alteration

Since the decrease in PRs was significant, we next looked at the loss of PRs after neoadjuvant therapy. Patients that were PR-positive at their baseline level and became PR-negative in the post-treatment sample were defined as having a PR loss. Among the 543 patients who were PR positive at baseline, 121 of them (22.2%) had a PR loss. While in Cohort-C, Cohort-E, and Cohort-EC, the rate of the PR loss was 16.6% (37/223), 29.8% (53/178), and 21.9% (31/142), respectively. Since the previous results suggested that a decrease in the PR Allred scores could be dominated by endocrine therapy, the PR loss was analyzed in the Cohort-E group by comparing the PR loss patients (*n* = 53) with those who remained PR positive after endocrine therapy (*n* = 125) (Table 4). While the pre-treatment PR Allred scores were significantly lower in the PR loss group, the ER Allred scores both in the pre-treatment and post-treatment samples showed no difference. Other biomarkers, including the Ki-67, EGFRs, and HER2, were also similar in the two groups. Interestingly, the length of endocrine therapy and the application of the aromatase inhibitor did not affect the frequency of PR losses. When the PR loss cases were analyzed in the Cohort-C and Cohort-EC groups, similar results were found except that, in the Cohort-C group, the PR loss was associated with lower ER Allred scores in both the pre-treatment and post-treatment samples (see Supplementary Tables S1 and S2). Our results showed that the lower the baseline PR Allred score was, the more likely it was that the breast cancer tissue would experience a PR loss due to therapy.

## 4. Discussion

A major finding in our study was that PR losses were more common than ER losses in all the NET, NCT, and NECT settings. Meanwhile, our results revealed that a PR loss after NET was strongly associated with a lower baseline PR score; however, we did not find a significant association between PR losses and Ki-67 reductions or ER losses (Table 3). Indeed, the baseline PR score, decrease of the PR score, and PR loss after systematic therapy had all been reported to be associated with the decreased disease-free survival in breast cancer, while the loss and decrease in ERs had not been [20,21]. However, since the prognostic role of low baseline PR expression was well established to be unfavorable in ER-positive and HER2-negative patients [21,22,23,24], it was highly possible that the decrease or loss of PRs after therapy was only a ‘passenger’ phenomenon without an additional prognostic value. PR losses in the metastasis lesions of breast cancer were also reported to be more frequent compared with ER losses [25]. The high frequency of PR losses during neoadjuvant therapies identified in our study warrants the further investigation of its role in the progress of ER-positive breast cancer. Indeed, contradictory evidence exists regarding the role of PRs in the carcinogenesis and progression of breast cancer [26,27,28,29,30,31]. Our results also showed that, in all three treatment groups, there was about 12% of patients who had an increase in Ki-67 in the post-treatment sample. In the ACOSOG Z1031 trial, it was found that 12% of the patients had an increase in Ki-67, which could potentially be due to treatment-resistant cells as indicated by the authors [3]. Interestingly, the percentage of patients with paradoxical increases in Ki-67 was similar among the three treatment groups, suggesting a shared subgroup of treatment-resistant patients.

Our study showed that the three types of neoadjuvant therapy had similar effects on the biomarker alteration of ER-positive/HER2-negative breast cancer (Figure 2 and Figure 3). More basic studies are needed to compare the molecular changes caused by NCT and NET for a better understanding of the optimal choice of therapy. A study showed that the functional ER pathway activity measured through mRNA expression decreased significantly two weeks after NET and did not decrease further during treatment [32]. Moreover, patients with a low baseline ER pathway activity were associated with a nonresponding status. For NCT, the expression of estrogen-related genes was also found to be downregulated after therapy [33]. Meanwhile, a recent study revealed that NET can also affect the stromal cell population by promoting the expansion of CD146 -/CDCP1 + stromal cells [34].

The strengths of this study included its large sample size, especially those of the NET and NECT groups, which enabled us to directly compare the biomarker alterations in these two groups with those in the NCT group with enough statistical analysis power. Also, our study provided unique data on biomarker alteration in the situation of extended NET use since the mean NET duration was longer compared with other studies. The major limitation of this study was due to its retrospective nature. The homogeneity of the studied population was not well controlled in terms of age, tumor size, and lymph node status.

## 5. Conclusions

Our study demonstrated that all three types of neoadjuvant therapies would cause a reduction in ER, PR, and Ki-67 expression. PR losses caused by neoadjuvant endocrine therapy were only significantly related to low baseline PR expression and were independent of Ki-67 reductions or ER losses.

## Figures and Tables

**Table 1:** Patient baseline information and treatment characteristics in the three treatment groups.

	Cohort-C	Cohort-E	Cohort-EC	*p*-Value
Total patients	633	314	247	
Age (years) ^1^	48.9 (9.9)	57.8 (10.5)	51.3 (10.3)	<0.001
Histology ^2^				0.492
IDC-NST	587 (92.7)	288 (91.7)	233 (94.3)	
Special subtype	46 (7.3)	26 (8.3)	14 (5.7)	
Histological grade ^2^				<0.001
I	88 (13.9)	62 (19.7)	35 (14.2)	
II	472 (74.6)	244 (77.7)	192 (77.7)	
III	73 (11.5)	8 (2.5)	20 (8.1)	
Surgery type ^2^				<0.001
BCS	242 (38.2)	197 (62.7)	138 (55.9)	
Mastectomy	391 (61.8)	117 (37.3)	109 (44.1)	
ER Allred score ^2^				<0.001
3/4/5	61 (9.6)	2 (0.6)	17 (6.9)	
6/7/8	572 (90.4)	312 (99.4)	230 (93.1)	
PR Allred score ^2^				0.001
0/2	63 (10.0)	13 (4.1)	15 (6.1)	
3/4/5	140 (22.1)	51 (16.2)	49 (19.8)	
6/7/8	430 (67.9)	250 (79.6)	183 (74.1)	
Ki-67 (%) ^1^	34.3 (22.5)	21.9 (16.9)	33.7 (21.8)	<0.001
NET duration (months) ^1^	N/A	9.9 (8.3)	8.5 (7.5)	0.043
Use of aromatase inhibitor ^2^				<0.001
No	633 (100.0)	29 (9.2)	40 (16.2)	
Yes	0 (0.0)	285 (90.8)	207 (83.8)	

^1^ Data are expressed as mean (sd); ^2^ data are expressed as n (%). Abbreviations: BCS represents breast-conserving surgery; ER represents estrogen receptor; PR represents progesterone receptor; NET represents neoadjuvant endocrine therapy; and IDC-NST represents invasive ductal carcinoma of no special type.

**Table 2:** Paired biomarker information of pre-therapy and post-therapy samples in the three groups.

	Cohort-C			Cohort-E			Cohort-EC		
	Before	After		Before	After		Before	After	
ER Allred score ^1^			*p* < 0.001			*p* < 0.001			*p* < 0.001
0/2		13 (5.3)			1 (0.5)			8 (5.4)	
3/4/5	17 (7.0)	14 (5.8)		2 (1.1)	6 (3.2)		7 (4.7)	6 (4.0)	
6/7/8	226 (93.0)	216 (88.9)		185 (98.9)	180 (96.3)		142 (95.3)	135 (90.6)	
PR Allred score			*p* < 0.001			*p* < 0.001			*p* < 0.001
0/2	20 (8.2)	47 (19.3)		9 (4.8)	60 (32.1)		7 (4.7)	38 (25.5)	
3/4/5	64 (26.3)	81 (33.3)		35 (18.7)	72 (38.5)		26 (17.4)	49 (32.9)	
6/7/8	159 (65.4)	115 (47.3)		143 (76.5)	55 (29.4)		116 (77.9)	62 (41.6)	
Ki-67(%), mean (SD)	32.4 (21.0)	14.0 (16.3)	*p* < 0.001	21.5 (17.7)	10.8 (14.7)	*p* < 0.001	31.3 (21.1)	13.7 (17.9)	*p* < 0.001
HER2			*p* < 0.001			*p* < 0.001			*p* < 0.001
0	48 (19.8)	74 (30.5)		29 (15.5)	65 (34.8)		32 (21.5)	51 (34.2)	
1	107 (44.0)	80 (32.9)		95 (50.8)	88 (47.1)		66 (44.3)	59 (39.6)	
2	88 (36.2)	87 (35.8)		63 (33.7)	31 (16.6)		51 (34.2)	37 (24.8)	
3		1 (0.4)						1 (0.7)	
Unknown		1 (0.4)			3 (1.6)			1 (0.7)	

^1^ Data are expressed as n (%) unless otherwise specified.

**Table 3:** Biomarker alteration in age and therapy length subgroups in the NET Cohort.

	Age ≤ 60 Years (*n* = 106)	Age > 60 Years (*n* = 81)	*p*-Value	NET Length ≤6 Months (*n* = 92)	NET Length >6 Months (*n* = 95)	*p*-Value
ER			0.68			0.2
Decrease (%)	40.6	37.0		30.9	45.3	
Even (%)	53.8	59.3		58.8	50.5	
Decrease (%)	5.7	3.7		5.2	4.2	
PR			0.42			0.53
Decrease (%)	80.2	84.0		79.4	80.0	
Even (%)	17.0	11.1		11.3	16.8	
Decrease (%)	2.8	4.9		4.1	3.2	
Ki-67			0.87			0.37
Decrease (%)	80.4	78.8		83.1	76.3	
Even (%)	6.9	8.8		4.5	10.8	
Decrease (%)	12.7	12.5		12.4	12.9	

**Table 4:** Clinicopathological characteristics of patients with and without PR-losses in Cohort-E.

	PR-Loss	PR-Preserved	*p*-Value
Total patients	53	125	
Age (years, mean (SD)) ^1^	58.6 (9.0)	57.4 (11.5)	0.503
Histology ^2^			0.028
IDC-NST	46 (86.8)	121 (96.8)	
Special subtype	7 (13.2)	4 (3.2)	
Histological grade ^2^			0.604
I	10 (18.9)	28 (22.4)	
II	41 (77.4)	95 (76.0)	
III	2 (3.8)	2 (1.6)	
Surgery type ^2^			0.285
BCS	37 (69.8)	75 (60.0)	
Mastectomy	16 (30.2)	50 (40.0)	
ER Allred score (pre) ^2^			1.000
3/4/5	1 (1.9)	1 (0.8)	
6/7/8	52 (98.1)	124 (99.2)	
ER Allred score (post) ^2^			0.274
0/2	1 (1.9)		
3/4/5	1 (1.9)	4 (3.2)	
6/7/8	51 (96.2)	121 (96.8)	
PR Allred score (pre) ^2^			0.001
3/4/5	19 (35.8)	16 (12.8)	
6/7/8	34 (64.2)	109 (87.2)	
PR Allred score (post) ^2^			< 0.001
0/2	53 (100.0)		
3/4/5		70 (56.0)	
6/7/8		55 (44.0)	
Ki-67 (%, pre) ^1^	22.9 (18.5)	20.6 (17.4)	0.428
Ki-67 (%, post) ^1^	8.4 (13.2)	10.4 (12.8)	0.370
EGFR (pre) ^2^			0.498
0	38 (71.7)	80 (64.0)	
1	3 (5.7)	13 (10.4)	
Unknown	12 (22.6)	32 (25.6)	
EGFR (post) ^2^			0.236
0	41 (77.4)	96 (76.8)	
1	12 (22.6)	23 (18.4)	
Unknown		6 (4.8)	
HER2 (pre) ^2^			0.439
0	7 (13.2)	20 (16.0)	
1	31 (58.5)	60 (48.0)	
2	15 (28.3)	45 (36.0)	
HER2 (post) ^2^			0.780
0	15 (28.3)	45 (36.0)	
1	27 (50.9)	59 (47.2)	
2	10 (18.9)	19 (15.2)	
Unknown	1 (1.9)	2 (1.6)	
NET duration (months) ^1^	10.1 (9.2)	9.1 (6.6)	0.378
Use of aromatase inhibitor ^2^			0.377
No	1 (1.9)	8 (6.4)	
Yes	52 (98.1)	117 (93.6)	

^1^ Data are expressed as mean (sd); ^2^ data are expressed as *n* (%).

## Data Availability

The data presented in this study are available on request from the corresponding author. The data are not publicly available due to restrictions by the ethical committee.

## Supplementary Materials

The following supporting information can be downloaded at https://www.mdpi.com/article/10.3390/life13010074/s1, Table S1: Comparison of clinicopathological characteristics between patients with PR-losses and patients with preserved PR statuses in Cohort-C group; Table S2: Comparison of clinicopathological characteristics between patients with PR-losses and patients with preserved PR statuses in Cohort-EC group.
