# Supplementary material for: Biomarker Alteration after Neoadjuvant Endocrine Therapy or Chemotherapy in Estrogen Receptor-Positive Breast Cancer
Source: Life (Basel). 2022 Dec 27;13(1):74. doi: 10.3390/life13010074 (PMC9866043; doi:10.3390/life13010074)
Supplement: Supplementary file 1 [file life-13-00074-s001.zip › life-1958154-supplementary.pdf]

Table S1. Comparison of clinicopathological characteristics between patients with PR-loss and patients with preserved PR status in Cohort-C group

	Negative	Positive	*P*
Age, mean (SD)	49.5 (9.8)	49.9 (9.8)	0.782
Histology			0.436
IDC-NST	35 (94.6)	165 (88.7)	
Special subtype	2 (5.4)	21 (11.3)	
Histological grade			0.194
I	3 (8.1)	32 (17.2)	
II	29 (78.4)	141 (75.8)	
III	5 (13.5)	13 (7.0)	
Surgery type			0.506
BCS	11 (29.7)	69 (37.1)	
Mastectomy	26 (70.3)	117 (62.9)	
ER Allred score			0.001
3-5	6 (16.2)	4 (2.2)	
6-8	31 (83.8)	182 (97.8)	
Post-ER Allred score			<0.001
0/2	4 (10.8)	4 (2.2)	
3/4/5	6 (16.2)	4 (2.2)	
6/7/8	27 (73.0)	178 (95.7)	
PR Allred score			<0.001
3/4/5	25 (67.6)	39 (21.0)	
6/7/8	12 (32.4)	147 (79.0)	
Post-PR Allred score			<0.001
0/2	37 (100.0)		
3/4/5		74 (39.8)	
6/7/8		112 (60.2)	
Ki-67, mean (SD)	37.3 (22.6)	30.5 (20.0)	0.066
PostKi-67, mean (SD)	14.2 (21.0)	12.4 (12.8)	0.507

EGFR			0.088
0	24 (64.9)	138 (74.2)	
1	5 (13.5)	8 (4.3)	
Unknown	8 (21.6)	40 (21.5)	
Post-EGFR			<0.001
0	19 (51.4)	130 (69.9)	
1	9 (24.3)	3 (1.6)	
Unknown	9 (24.3)	53 (28.5)	
HER2			0.528
0	5 (13.5)	38 (20.4)	
1	16 (43.2)	82 (44.1)	
2	16 (43.2)	66 (35.5)	
Post-HER2			0.416
0	11 (29.7)	56 (30.1)	
1	8 (21.6)	65 (34.9)	
2	18 (48.6)	63 (33.9)	
3		1 (0.5)	
Unknown		1 (0.5)	

Table S2. Comparison of clinicopathological characteristics between patients with PR-loss and patients with preserved PR status in Cohort-EC group

	Negative	Positive	*P*
Age, mean (SD)	47.8 (9.8)	51.6 (10.1)	0.064
Histology			0.035
IDC-NST	26 (83.9)	107 (96.4)	
Special subtype	5 (16.1)	4 (3.6)	
Histological grade			0.692
I	4 (12.9)	19 (17.1)	
II	25 (80.6)	88 (79.3)	
III	2 (6.5)	4 (3.6)	
Surgery type			0.139
BCS	22 (71.0)	60 (54.1)	
Mastectomy	9 (29.0)	51 (45.9)	
ER Allred score			1.000
3/4/5	1 (3.2)	2 (1.8)	
6/7/8	30 (96.8)	109 (98.2)	
Post-ER Allred score			0.291
0/2	2 (6.5)	2 (1.8)	
3/4/5	2 (6.5)	4 (3.6)	
6/7/8	27 (87.1)	105 (94.6)	
PR Allred score			0.045
3/4/5	10 (32.3)	16 (14.4)	
6/7/8	21 (67.7)	95 (85.6)	
Post-PR Allred score			<0.001
0/2	31 (100.0)		
3/4/5		49 (44.1)	
6/7/8		62 (55.9)	
Ki-67, mean (SD)	33.6 (20.7)	29.8 (20.1)	0.362
PostKi-67, mean (SD)	11.8 (19.9)	12.1 (14.1)	0.917

EGFR			0.894
0	21 (67.7)	76 (68.5)	
1	3 (9.7)	8 (7.2)	
Unknown	7 (22.6)	27 (24.3)	
Post-EGFR			0.901
0	23 (74.2)	86 (77.5)	
1	1 (3.2)	4 (3.6)	
Unknown	7 (22.6)	21 (18.9)	
HER2			0.221
0	6 (19.4)	24 (21.6)	
1	18 (58.1)	46 (41.4)	
2	7 (22.6)	41 (36.9)	
Post-HER2			0.155
0	13 (41.9)	35 (31.5)	
1	13 (41.9)	45 (40.5)	
2	4 (12.9)	30 (27.0)	
3		1 (0.9)	
Unknown	1 (3.2)		
NET duration (months), mean (SD)	9.8 (9.0)	8.3 (7.1)	0.324
Use of aromatase inhibitor			0.276
No	2 (6.5)	18 (16.2)	
Yes	29 (93.5)	93 (83.8)	
